# Beyond the incision: assessing bleeding risks in early aspirin administration post coronary artery bypass grafting

**DOI:** 10.1007/s11845-025-03990-9

**Published:** 2025-06-23

**Authors:** Laura M. Staunton, Sarah Early, Michael Tolan, Vincent K. Young, Saleem M. Jahangeer

**Affiliations:** Cardiothoracic Surgery, St James’s Hospital, James Street, Dublin 8, D08NHY1 Dublin, Ireland

**Keywords:** Antiplatelet drugs, Antithrombotic drugs, Aspirin, Coronary artery bypass grafting (CABG)

## Abstract

**Background:**

Guidelines recommend early aspirin loading (150–325 mg) within 6 h of coronary artery bypass grafting (CABG), which improves patency of vein grafts. Due to bleeding concerns, this is not standard practice.

**Aims:**

The aim of this study is to review early aspirin administration, within 6 h of CABG, to determine if there is an increased risk of bleeding.

**Methods:**

Bleeding risk was evaluated in 160 patients, undergoing CABG procedure from January 2022 to February 2023. Patients were divided into two groups: those that received 300 mg of aspirin within 6 h (group 1) and those that did not (group 2). Drainage output from different timepoints, red cell, platelet transfusion rates, pericardial effusion, and re-exploration for bleeding were reviewed. Statistical analysis was performed using STATA/BE version 18.0. Significance was demonstrated when *p* value < 0.05.

**Results:**

Mean output drainage at 24 h was 695.7 mL for group 1 and 712.7 mL for group 2. Considering 11 timepoints, there were no significant difference between groups (*p* values = 0.731–0.117). Transfusion rates for red cells (*p* = 0.734) and platelets (*p* = 0.274), re-exploration for bleeding (*p* = 0.694), and pericardial effusion rates (*p* = 0.472) also showed no statistical difference.

**Conclusions:**

A comprehensive review of drainage output, red cell, platelet transfusion, re-exploration for bleeding, and pericardial effusion rates was performed, post early aspirin administration. Aspirin (300 mg) given within 6 h of CABG surgery did not lead to increased bleeding and associated complications.

## Introduction

Platelet aggregation causing graft thrombosis is the main cause of early graft failure, especially within 1 year of surgery [1]. ACC/AHA, EACTS guidelines recommend early aspirin loading within 6 h of coronary artery bypass grafting (CABG), which improves patency of vein grafts [1–3]. The ideal approach is administration of 150–325 mg (termed medium dose) aspirin within 6 h of CABG [1–9]. The efficacy reduces the later aspirin is administered, with no benefit seen after 48 h [1]. Due to concerns for increased post-operative bleeding, this is not standard practice amongst cardiac surgeons.

This study aims to provide an observational retrospective review of bleeding between patients that received early aspirin and those that did not. Our hypothesis is that no significant difference will be noted between groups. This study will hope to provide data, facilitating cardiac surgeons decision-making, and provide evidence to enable a change in practice, regarding use of early aspirin post CABG.

## Materials and methods

**Fig. 1:**
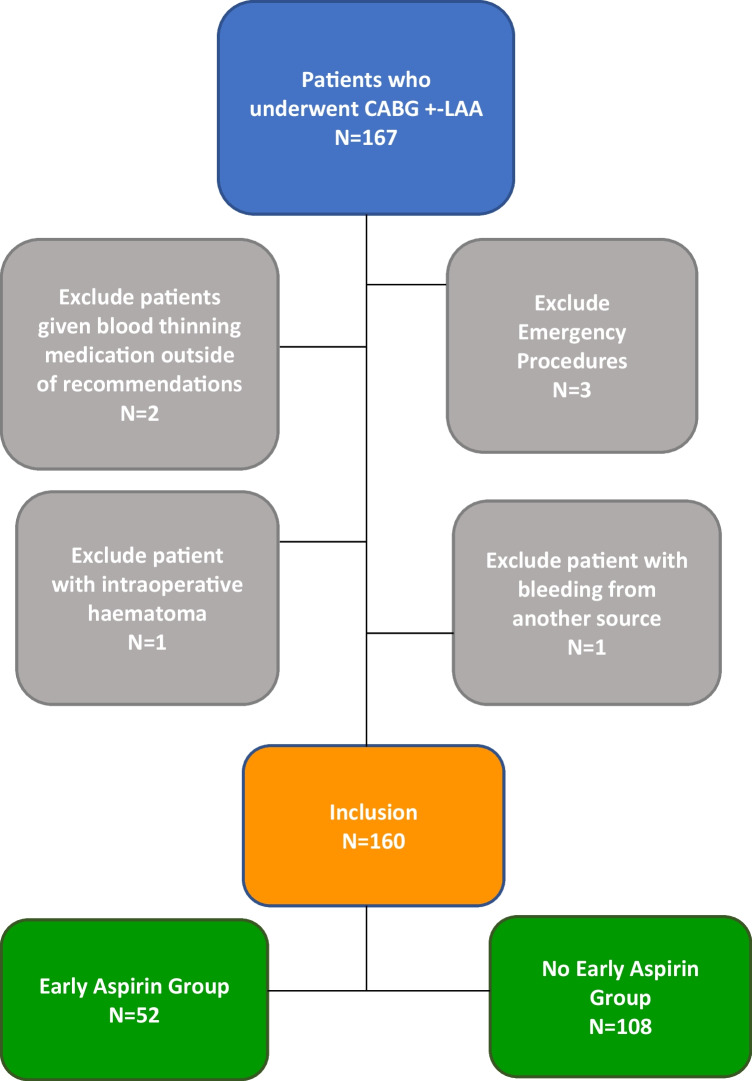
Selection of study participants. Inclusion criteria patient who underwent CABG + -LAA in the selected study period. Exclusions included patients on dual anti-platelets (DAPT)/anti-coagulation that were not discontinued pre-operatively as per guidelines, excessive bleeding from another sources and emergency procedures

A total of 167 patients were identified who underwent CABG from January 2022 until February 2023 via a retrospective observational study, performed at a single institution in Ireland. Cardiopulmonary bypass procedure was performed on all patients. Temporary epicardial electrodes for external pacing are placed intra-operatively as standard and removed on day 3. Exclusions included patients with bleeding conditions/coagulapathies including von Willibrand disease and factor V Leiden and patients on dual anti-platelets (DAPT)/anti-coagulation that were not discontinued pre-operatively as per guidelines [2] and emergency procedures. See Fig. 1 for details. Demographics as well as EuroScore II, which stratifies risk, when considering a patient for cardiac surgery, were collected to demonstrate comparability between groups [10].

Two groups were considered: those that received 300 mg of aspirin within 6 h of CABG (Group 1), compared to the second group, that did not receive early aspirin (Group 2). The primary outcome is volume output from drains (mL). Drains are placed intra-operatively post bypass. Drainage volumes were measured at 11 timepoints until drain removal, due to aspirin’s inhibitory effect on platelet aggregation and therefore potential increased bleeding risk, which can continue for several days [1–3]. Secondary outcomes considered are red cell and platelet transfusion rates, evidence of pericardial effusion (as determined on post-operative transthoracic echo (TTE) and re-exploration for bleeding (re-sternotomy). Pericardial effusions deemed present when > 1 cm, as this is deemed extra-ordinary blood loss. The exposure variable is early aspirin (300 mg) given within 2–6 h of CABG. Aspirin is given orally if the patient was extubated or rectally if not. Follow-up was performed until discharge.

Categorial variables were analysed using Pearson chi-squared. Wilson rank sum test or Student’s *t*-test was performed on continuous data, using STATA/BE version 18. Regression analysis was performed considering confounders: gender, ejection fraction (EF), operation duration, estimated intra-operative blood loss, post-op DAPT, or therapeutic low molecular weight heparin (tLMWH). Estimated intra-operative blood loss was documented at time of procedure. The other covariates that were considered are baseline drain output prior to giving early aspirin (2 h) and urgency of procedure. The confounders were chosen for the following reasons: Male gender is a risk factor for increased post-operative bleeding as examined in numerous studies [11–13]. EF is indicative of heart failure as per 2021 European Society of Cardiology (ESC) heart failure guidelines [14]. Patients with reduced ejection fraction are prone to pleural effusions. As drains can be placed intra-operatively in the pleural space, patients with reduced EF can have increased output from drains [11–13]. Operation duration, number of grafts, coronary artery bypass time, and cross-clamp times were all reviewed. These are intrinsically related; therefore, operation duration was only considered in the multi-variate regression analysis. When operation duration exceeds 3 h, increased blood loss can occur [11–13]. If estimated blood loss is high, this can impact transfusions rates, as well as increased heparin doses and coagulopathic abnormalities, which can increase blood loss [13]. DAPT and anti-coagulation can led to increased blood loss [1], therefore, the post-operative day of administration was collected. tLMWH is not given alongside DAPT in our facility. Elective and urgent procedures are included in this study. Urgent procedures are performed in cases, for example, post myocardial infarction, critical coronary stenosis, and unstable angina. This higher risk procedure, with or without potential cardiac injury, is an important covariate to consider.

For secondary outcome analysis, red cell and platelet transfusion rates were analysed separately in a univariable linear regression analysis and multivariable linear regression analysis including predictor variables as above and pre-operative haemoglobin. Patients undergoing cardiac surgery with high haemoglobin have risk of excessive bleeding [11]. Patients with a lower pre-operative haemoglobin are shown to have increased transfusion rates [11–13]. Pericardial effusion rates and re-exploration for bleeding consist of binary data; a univariable and multivariable logistic regression analysis was performed using the above confounders.

## Results

### Missing data

There were minimal missing data sets across the variables. There was no missing data from the primary outcome or exposure variable. There was no loss to follow-up, as all data points from the drain output at each timepoint was complete. As is acceptable in literature if the missing data is < 5%, it is deemed “inconsequential” and the rest of the data point will be included, without a statistical analysis for missing data performed [15]. If the missing data is > 5%, then depending on the data type and pattern of data, depending on the amount of data missing, a maximum likelihood or multiple imputation was performed [15]. Multiple imputation was used in this study.

### Pre-operative analysis

A comparison of the patient demographics and pre-operative statistics between groups can been seen in Table 1. There was a significant difference (*p* = 0.009) between ages from the two groups. There was a significant difference (*p* = 0.001) between groups for urgency of procedure, with the more urgent cases incorporated into the no aspirin group (73% vs 49%). All other differences were insignificant.

**Table 1:** Patient demographics and pre-operative characteristics

Characteristic	Early aspirin (*n* = 52) *N* (%/CI)	No early aspirin (*n* = 108) *N* (%/CI)	*P* value
Female	3 (6)	12 (11)	0.278
Male	49 (94)	96 (89)	
Age (mean, CI^1^) years	60.8 (58–63.6)	65.1 (63.3–66.9)	0.009
EuroScore II % (mean, ± CI^1^)	1.74 (1.09–2.38)	2.02 (1.65–2.39)	0.478
Urgency of procedure			
Elective	30 (58)	29 (27)	0.001
Urgent	22 (42)	79 (73)	
History of DAPT^2^			
Yes	23 (44)	49 (45)	0.892
No	29 (56)	59 (55)	
Anticoagulation^2^			
Yes	14 (27)	12 (11)	0.011
No	38 (73)	96 (89)	
Ejection fraction (mean, ± CI^1^) ^3^	40.5 (48.1–52.9)	49.5 (47.7–51.3)	0.530
Normal (> 50%)^3^	38 (73)	73 (69)	0.584
Mildly Reduced (41–49%)^3^	6 (11.5)	8 (8)	0.386
Moderately Reduced (35–40%)^3^	6 (11.5)	14 (13)	0.799
Severely reduced (< 35%)^3^	2 (4)	12 (11)	0.128
Bleeding conditions	0	0	N/A
Cardiac surgery history	0	0	N/A
Pre-op haemoglobin (mean, ± CI^1^) g/dL	13.13 (12.7–13.5)	13.3 (13.1–13.6)	0.654

^1^95% confidence interval

^2^Only participants that anticoagulation and/or DAPT were stopped as per guidelines pre-op were included

^3^2021 ESC heart failure guidelines: > 50%—normal EF, 41–49%—mildly reduced EF, 35–40%—moderately reduced EF, and < 35%—severely reduced EF^16^

### Analysis

All patients in both groups received one arterial graft (left internal mammary artery), alongside venous grafts from saphenous vein harvesting. Median operation duration was 302 min (IQR: 268–343) in the group 1 and 272 (IQR 230–306) minutes in group 2. The difference was significant (*p* = 0.001). Significant differences are also noted in cross clamp and bypass times (*p* = 0.001). The median estimated intraoperative blood loss was 525 mL (IQR 425.576.5) in group 1 and 512.5 mL (365–690) in group 2, showing no significant difference (*p* = 0.129). The majority of patients in each group did not have red cell transfusion in theatre, 75% in early aspirin group and 69% in no early aspirin group. There was no significant difference (*p* = 0.410) between groups. Majority of patients in each group did not require platelets, 85% in early aspirin group and 77% in no aspirin group, with no significance difference noted (*p* = 0.630). Refer to Tables 2 and 3 for details.

**Table 2:** Intra-operative characteristics

Characteristic	Early aspirin (*n* = 52) *N* (%/CI)	No early aspirin (*n* = 108) *N* (%/CI)	*P* value
Operation duration, mins (median, IQ range)	302 (268–343)	272 (230–306)	0.001
Cross clamp time, mins (median, IQ range)	78.5 (58.5–94)	56 (42–64)	0.001
Bypass time, mins (median, IQ range)	111 (81.5–131)	92 (69–105)	0.001
Estimated Intraoperative blood loss, mL (median, IQ range)	525 (425.5–765)	512.5 (365–690)	0.129
Number. of grafts			
1	6 (12)	6 (5)	0.145
2	15 (29)	26 (24)	
3	24 (46)	58 (54)	
4	7 (13)	18 (17)	
RCC theatre			
o None	39 (75)	74 (69)	0.410
o One Unit	5 (10)	13 (12)	
o 2–5 units	8 (15)	21 (19)	
Platelet theatre			
o None	44 (85)	83 (77)	0.623
o One unit	2 (4)	14 (13)	
o 2–5 units	11 (11)	6 (10)	

^1^95% confidence interval

**Table 3:** Post-operative characteristics

Characteristic	Early aspirin (*n* = 52) *N* (%/CI)	No early aspirin (*n* = 105) *N* (%/CI)	*P* value
Hb on discharge (mean, ± CI^1^) g/dL	10.2 (9.7–10.5)	9.8 (9.6–10)	0.118
Day drain out (mean, ± CI^1^)	2.42 (2.1–2.7)	3 (2.8–3.2)	0.001
Time aspirin given (mean, ± CI^1^) hours	5 (4.6–5.2)	0	N/A
Aspirin day 1 (75 mg)	51 (98)	105 (97)	0.673
DAPT free	9 (17)	63 (58)	0.001
DAPT Days 1–2	43 (83)	0	
DAPT Days 3–7	0	45 (42)	
Enoxaparin free	45 (87)	105 (97)	
tLMWH Days 1–2	7 (13)	0	0.009
tLMWH Days 3–7	0	3 (3)	
Mortality rates	0	1 (1)	0.462

^1^95% confidence interval

### Drain output

Data was collected and analysed at 11 timepoints. There was no significant difference between groups at any given timepoint considering univariable and multivariable regression analysis. Two hours was utilised in the analysis as a baseline. Refer to Tables 4 and 5 for mean drainage at each timepoint, which was collected as total output. There was no significant difference at any timepoint. Follow-up was performed, until drain removal.

**Table 4:** Comparison of outcome variables

Outcome variable	Early aspirin (*n* = 52) *N* (%/CI)	No early aspirin (*n* = 105) *N* (%)	*P* value	Coefficient/odds ratio
Drain output (mL) mean (CI)				
o 2 h	129 (112–145.9)	117.7 (100–135.3)	0.406	11.3
o 4 h	208.9 (185.4–232.4)	191.9 (167.3–216.7)	0.375	16.9
o 6 h	266.8 (239–295)	264.8 (234–295)	0.932	2.00
o 8 h	315.5 (284.3–346.7)	322.9 (274.1–371.7)	0.835	− 7.34
o 10 h	357.3 (321.8–393.9)	363.8 (309.9–417.7)	0.870	− 6.45
o 12 h	407.3 (366.4–448.3)	404.7 (347.7–461.8)	0.951	2.6
o 18 h	571.9 (518.1–625.7)	555.1 (489.1–621.1)	0.736	16.76
o 24 h	712.7 (644.8–780.5)	695.7 (617–774.)	0.777	16.9
o 36 h	857.8 (777.3–938.2)	829.2 (744.9–913.5)	0.662	28.54
o 48 h	1050.7 (943.5–1157.9)	962.3 (867.6–1056.9)	0.247	88.4
o Total	1224 (981–1446)	1279 (1116–1442)	0.701	− 55.08
RCC				
Units (mean, CI)	0.40 (0.20–0.60)	0.40 (0.23–0.56)	0.991	− 0.001
o None	38 (73)	82 (76)		
o One unit	6 (12)	16 (15)		
o 2–5 units	8 (15)	10 (9)		
Platelets				
Mean	0.05 (− 0.05–1.5)	0.11 (0.01–0.2)	0.447	− 0.05
o None	102 (94)	51 (98)		
o One unit	2 (2)	0		
o 2–5 units	4 (4)	1 (2)		
Pericardial effusion (> 1 cm)	3 (5)	1 (1)	0.130	5.836
Re-sternotomy	1 (2)	2 (2)	0.953	0.930

^1^95% confidence interval

**Table 5:** Multivariate analysis on the primary outcome variable at multiple timepoints

Timepoint	Coefficient	Std err	*P* value	95% confidence interval
4 h	− 2.790	8.114	0.731	18.81–13.24
6 h	− 21.595	13.707	0.117	− 48.67–5.48
8 h	− 38.339	27.481	0.175	− 92.64–15.96
10 h	− 41.7464	29.9	0.166	− 101.02–17.53
12 h	− 40.948	28.602	0.154	− 97.45–15.55
18 h	− 28.92	39.557	0.466	− 107.09–49.24
24 h	− 30.141	43.549	0.490	− 116.171–55.89
36 h	− 21.551	58.181	0.712	− 136.512–93.42
48 h	30.217	65.899	0.647	− 99.967–160.4
Drain out	− 135.074	132.121	0.308	− 396.077–125.93

### Transfusion

Red cell transfusion rates (*p* = 0.991, OR − 0.001, *p* = 0.734, CI − 0.359–0.254) and platelet transfusion rates (*p* = 0.447, OR − 0.05, *p* = 0.274, CI − 0.285–0.082) showed no significant difference when univariable or multivariable analysis was performed.

### Pericardial effusion and re-sternotomy

**Fig. 2:**
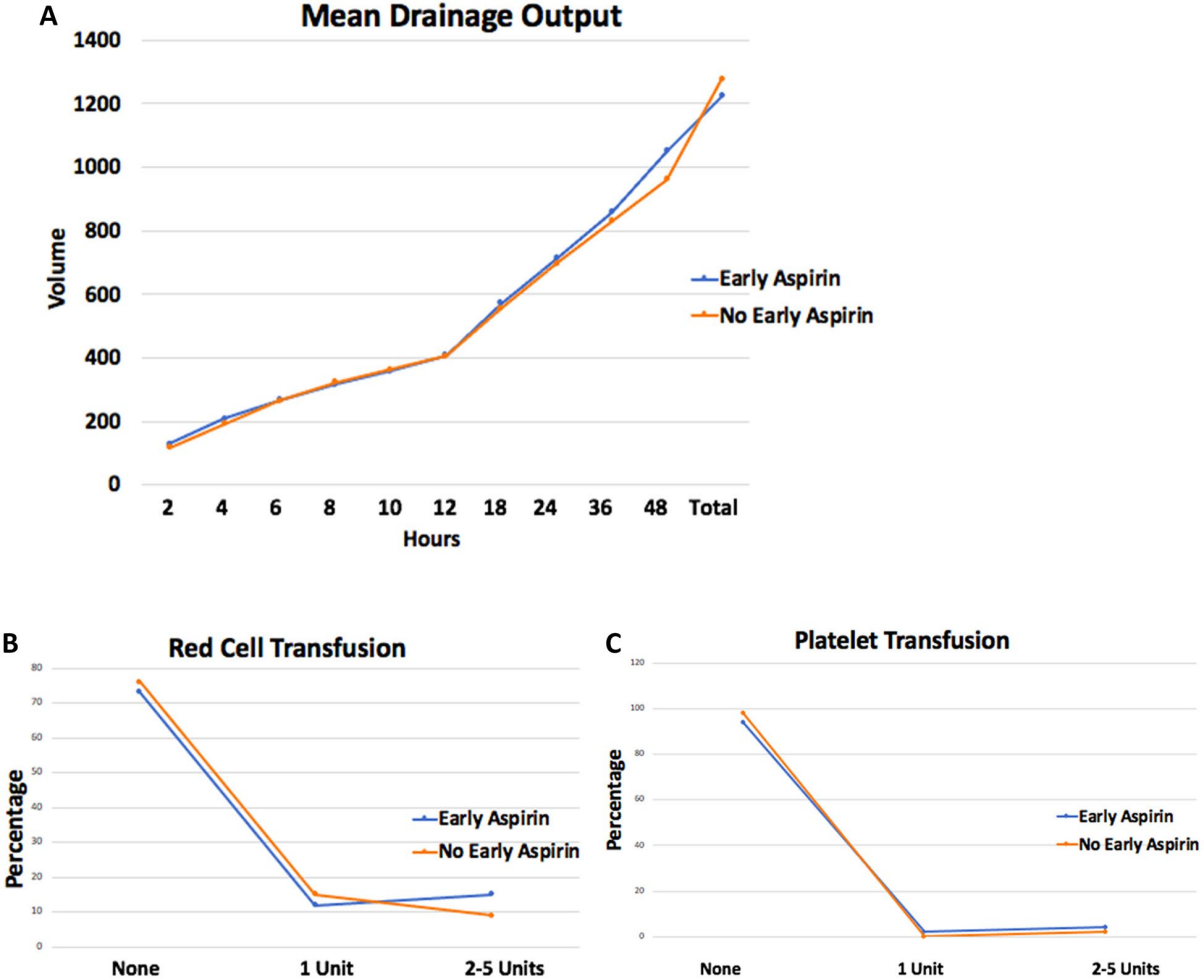
Comparison of primary and secondary endpoints. **A** Plot showing mean volume of drainage at different timepoints until drain was removed. **B** Plot showing red cell transfusion percentage rates when none given, 1 unit and 2–5 units. **C** Plot showing platelet transfusion, percentage rates when none given, 1 units and 2–5 units. All compare early aspirin administration and no early aspirin given

Pericardial effusion rates (*p* = 0.090, OR 5.836; *p* = 0.472, CI − 6.391–13.794) and re-exploration for bleeding (*p* = 0.953, OR 0.930; *p* = 0.694, CI − 3.186–4.785) were not significant on regression analysis. Refer to Tables 4 and 6 and Fig. 2 for details.

**Table 6:** Multivariate analysis on secondary outcomes

Timepoint	Coefficient	Std err	*P* value	95% confidence interval
Red cell	− 0.0527	0.155	0.734	− 0.359–0.254
Platelets	− 0.102	0.093	0.274	− 0.285–0.082
Re-exploration	0.799	2.033	0.694	− 3.186–4.785
Pericardial effusion	3.701	5.149	0.472	− 6.391–13.794

## Discussion

This study reveals the administration of 300 mg aspirin, within 6 h of coronary artery bypass grafting, showed no increased bleeding risk within the limitations of this study.

The descriptive analysis showed a number of statistically significant variables between the group 1 and group 2. The significant difference in age (*p* = 0.009) is likely due to the fact that the group 2 had a larger number of individuals over 70 (36% and 25%). Age is not a covariate for bleeding [11–13]. Analysis of urgency of procedure revealed a significant difference (*p* = 0.001) as the group 2 had 73% urgent cases, whereas the early aspirin group had more elective cases (58%). This variable is adjusted for in the multivariable analysis. CABG took longer in group 1 compared to group 2 (*p* = 0.001); cross clamp time (0.001) and CPB time (0.001) were significantly different. The statistical difference in overall operating time, cross-clamp and CPB time, may be due to difference in surgical technique (e.g., single clamp technique, longer harvest times due to arterial grafting). A longer operation duration can be a factor causing excessive bleeding [11–13]. However, increased operation duration has been shown to cause excessive blood loss, only when extended beyond 3 h [11–13]. The mean time of group 1 (111 min) and group 2 (92 min) did not exceed 3 h. Therefore, this difference, as well as the differences in surgical technique, does not have an impact in bleeding rates in our cohort. Operation time is adjusted for in multivariable analysis.

Eleven timepoints were analysed for this study. The total output was measured on drain removal. Drain was removed when < 300 mL in 24 h. The 2-h timepoint was used as baseline as all early aspirin was given between 2 and 6 h post CABG (mean = 5 h). The 4- and 6-h timepoint was considered important as it was approximately time of exposure. At each time point, no statistical significance for drain output can be seen between groups, as per Tables 4 and 5.

The transfusion rates for red cells (*p* = 0.991, OR − 0.001; *p* = 0.734, CI − 0.359–0.254) and platelets (*p* = 0.447, OR − 0.05; *p* = 0.274, CI − 0.285–0.082) were not significantly different between groups before or after adjusted regression analysis. Neither were there significant differences between re-sternotomy (*p* = 0.953, OR 0.930; *p* = 0.694, CI − 3.186–4.785) or pericardial effusion rates (*p* = 0.090, OR 5.836; *p* = 0.472, CI − 6.391–13.794). Post-operatively anti-coagulation and DAPT was initiated earlier in group 1 patients on day 1 (*p* = 0.001, *p* = 0.009). Anti-coagulation has the potential to confound bleeding rates between groups; however, no difference in bleeding was noted between groups.

Current guidelines recommend optimal dosage and timeline of early aspirin to be 150–325 mg, given within 6 h of CABG surgery or when bleeding subsides [1–3]. There is continued belief that aspirin’s anti-platelet activity, when given post-operatively, will cause excessive bleeding in the post-operative period. Some of the earlier RCTs show increased blood loss with aspirin [4, 7, 8]. One specifically published by the VA in 1988 concluded that while there was an improvement in graft patency with aspirin, aspirin caused increased post-operative bleeding and re-operation rates [5]. However, aspirin was given 12 h pre-operatively and re-commenced 6 h post CABG [5]. The authors acknowledge at the time that the pre-operative dose is likely the cause of the increased bleeding [5]. Subsequent trials have proved that pre-operative aspirin at medium or high doses causes excessive post-operative blood loss, transfusion, and re-operation [8].

Individual randomised trials and meta-analysis reviewing administration of post-operative aspirin within 6 h show no significant difference in bleeding post exposure [4, 9]. Gavaghan et al. published a randomised trial in 1991 showing that early aspirin within 1 h of CABG improved early and late graft patency [4]. Blood loss from drains, re-operation, and transfusion rates were also reviewed, and no significant difference was found between early aspirin and placebo [4]. A meta-analysis performed by Fremes et al. in 1993, reviewing 17 RCTs again showing early aspirin, had a protective effect on graft patency, with no significant difference in blood loss in the post-operative period [8]. These trials reviewed blood loss from drains at a single timepoint. There is no other study reviewing drain output at different timepoints after early aspirin was given. Multiple timepoints were considered due to the different confounders that exist for bleeding post CABG. As early aspirin is recommended within 6 h or when bleeding subsides, it is also important to be aware of the mean drain output before and after early aspirin is administered. These trials also consider mean red cell transfusion and re-exploration rates. Our study reviews red cell and platelet transfusion, re-exploration for bleeding alongside pericardial effusion rates. Also, previous studies only consider hypothesis testing and do not use adjusted regression analysis [4, 9, 16, 17].

Post-operative aspirin dosing and timing is different in literature. Gavaghan et al. utilized 325 mg aspirin within 1 h of CABG [4]. Goldman et al. gave 325 mg pre-operative and post-operative aspirin [5]. Khan et al. compare 150 mg aspirin at 6 h or 12 h [18]. Gukop et al. review the literature on post-operative bleeding with early aspirin [1, 16]. They discovered 11 relevant studies, and their recommendation was to proceed to early aspirin administration at 6 h. Most trials were performed with the primary objective, of improving graft patency. As well as improving graft patency, aspirin use within 48 h of surgery (dose between 80 and 650 mg) has been shown to have a positive impact on mortality and ischemic events, including decreased incidence of MI (48%), stroke (50%), bowel infarction (62%), and renal failure (72%) [1, 17].

The different dosing regimens of aspirin in studies, the use of dual antiplatelets and anti-coagulation in the perioperative and post-operative period, and the lack of focused studies with adequate statistical modelling all create confusion in decision-making around use of early aspirin in the post-operative period. A published audit in Liverpool reviewed reasons why poor compliance to early aspirin exists [19]. They cited that lack of awareness, alongside cultural based practices, was barriers to compliance in their institution [19]. A multi-disciplinary approach with medical, nursing, and pharmacy staff were key to process improvement, with 182% increase in early aspirin administration post CABG, after the audit process [19]. Gukop et al. consider the importance of setting a bleeding threshold, whereby early aspirin would not be given [1, 16]. This would then avoid unnecessary delay in administration [1, 16].

### Limitations of the study

This study shows bias toward male gender. Group 1 consisted of 94% and group 2 of 89% of male participants. In studies, the male gender is an independent risk factor for increased bleeding post cardiac surgery [1, 13]. Even though there no significant difference between male gender in this research (*p* = 0.278), a study with even numbers of males and females may give more accurate drain output findings. Not all patients in this study underwent a post-operative TTE. TTEs were performed only if there was a clinical suspicion of pericardial effusion. A number of confounding variables were considered, refer to the “Materials and methods” section. Bias as part of a retrospective study should also be considered. Sample sizes are small with 52 in early aspirin group and 108 in no aspirin group.

## Conclusions

This focused study with a comprehensive analysis on all aspects of bleeding including different intervals of drain output, red cell and platelet transfusion rates, re-exploration for bleeding, and pericardial effusion, alongside a comprehensive literature review enhances the literature already available. Aspirin (300 mg) given within 6 h of CABG surgery did not show an increase in post-operative bleeding in our study.

## Data Availability

The data underlying this article will be shared on reasonable request to the corresponding author.
